# Cytokine engineered NK-92 therapy to improve persistence and anti-tumor activity

**DOI:** 10.7150/thno.79942

**Published:** 2023-03-05

**Authors:** Hyun Young Shin, Seil Jang, Hyeong Jung Woo, Jae-Hee Chung, Woon-Hae Kim, Dongoh Kim, Minju Kang, Yujin Lim, Omer Habib, Jungmin Lee, Sohae Yang, Dae Hee Lee, Minseok S. Kim

**Affiliations:** 1 CTCELLS Inc., 216, Gaepo-ro, Gangnam-gu, Seoul, 06307, Republic of Korea; 2 Department of New Biology, DGIST, Daegu, 42988, Republic of Korea; 3 Translational Responsive Medicine Center (TRMC), DGIST, Daegu 42988, Republic of Korea; 4 New Biology Research Center (NBRC), DGIST, Daegu 42988, Republic of Korea

**Keywords:** natural killer cell, membrane-bound protein (MBP), interleukin-2, self-activation, tumor-infiltrating lymphocytes, microwell array chip

## Abstract

Natural killer (NK) cells are an attractive cell source in cancer immunotherapy due to their potent antitumor ability and promising safety for allogenic applications. However, the clinical outcome of NK cell therapy has been limited due to poor persistence and loss of activity in the cytokine-deficient tumor microenvironment. Benefits from exogenous administration of soluble interleukin-2 (IL-2) to stimulate the activity of NK cells have not been significant due to cytokine consumption and activation of other immune cells, compromising both efficacy and safety.

**Methods:** To overcome these drawbacks, we developed a novel membrane-bound protein (MBP) technology to express IL-2 on the surface of NK-92 cells (MBP NK) inducing autocrine signal for proliferation without IL-2 supplementation.

**Results:** The MBP NK cells exhibited not only improved proliferation in IL-2 deficient conditions but also stronger secretion of cytolytic granules leading to enhanced anti-tumor activity both *in vitro* and *in vivo*. Furthermore, the experiment with a spheroid solid tumor model exhibited enhanced infiltration by MBP NK cells creating higher local effector-to-target ratio for efficient tumor killing. These results suggest MBP technology can be an effective utility for NK-92 cell engineering to increase anti-tumor activity and reduce potential adverse effects, providing a higher therapeutic index in clinical applications.

## Introduction

As the conventional anticancer drugs used for the past several decades have caused severe side effects and drug resistance, immunotherapy, which aims to enhance and redirect the immune system against tumor cells, is drawing attention as a novel anti-cancer treatment with improved efficacy and safety. Cellular immunotherapy agents such as Novartis' Kymriah, a chimeric antigen receptor (CAR)-T cell, have attracted significant interest after receiving FDA approval based on excellent clinical results for the treatment of refractory hematological cancers 1. However, the currently commercialized CAR-T cells require extraction and ex vivo expansion of the patient's own immune cells to avoid the risk of graft-versus-host diseases (GvHD). In addition, such an autologous method significantly increases therapeutic costs, creating challenges in mass production but the risk of cytokine release syndrome (CRS) and neurotoxicity still remain unsolved 2. On the other hand, natural killer (NK) cells, which are freer from the risk of GvHD, CRS, and neurotoxicity, have been proposed as a safer alternative for targeted therapy of cancer 3.

To transform NK cells into an applicable medication, efficient cell expansion strategy for mass production is first required. Various sources of NK cells have been evaluated for scalable expansion and NK-92, an immortalized clonal cell line from a patient with NK-cell lymphoma, has been one of the promising candidates. Easy expansion and clonal outgrowth of NK-92 3 requiring only common laboratory media (e.g. RPMI 1640 or MEMα) with IL-2 as a supplement could eliminate challenges found in other sources of NK cells such as derivation from healthy donors or differentiation from stem cells. Only small portion of circulating lymphocytes are NK cells making the collection process inconvenient and limited while their genetic modification requires viral gene delivery which is further complicated by the low proliferation level of NK cells. Differentiation into homogenous and functional NK cells from stem cells is cost-inefficient and still is a technical difficulty. These conveniences of NK-92 have led to intensive exploration in pre-clinical research some of which have been extended to clinical studies 4-8.

Nonetheless, NK based therapy still faces technical hurdles particularly low cellular activity *in vivo*. While interleukin-2 (IL-2), which is approved for its usage in clinical research 9, stimulates the proliferation and survival of NK cells 10, it is down-regulated in cancer patients 11. Thus, co-treatment of IL-2 with NK cells has been the widely accepted strategy in the clinical trials of NK cell therapy 12, 13. Still, exogenous IL-2 stimulates other regulatory immune cells such as Treg cells that inhibit the activity of transferred NK cells 14 and elevated serum concentration of soluble IL-2 receptor in cancer patients 11 may act as an antagonist against injected IL-2, reducing the percentage of activated NK cells. The short half-life (< 30 minutes) of IL-2 in a body also requires frequent injections that may cause side effects such as capillary leak syndrome 15. To avoid such IL-2-related disadvantages, NK-92MI cell, a virally modified IL-2-secreting NK-92 cell line, has been developed 6, 16, but the control of IL-2 dosage would become more complicated in this strategy and the paracrine stimulation by secreted IL-2 can still cause the above-mentioned side effects. In summary, co-treatment of soluble IL-2 inevitably accompanies biological effects without therapeutic benefits due to unintended stimulations of other cells, and hence, it is necessary to develop a technique that can exclusively deliver proliferation signals of IL-2 to NK cells at a single cell level.

**Figure 1:**
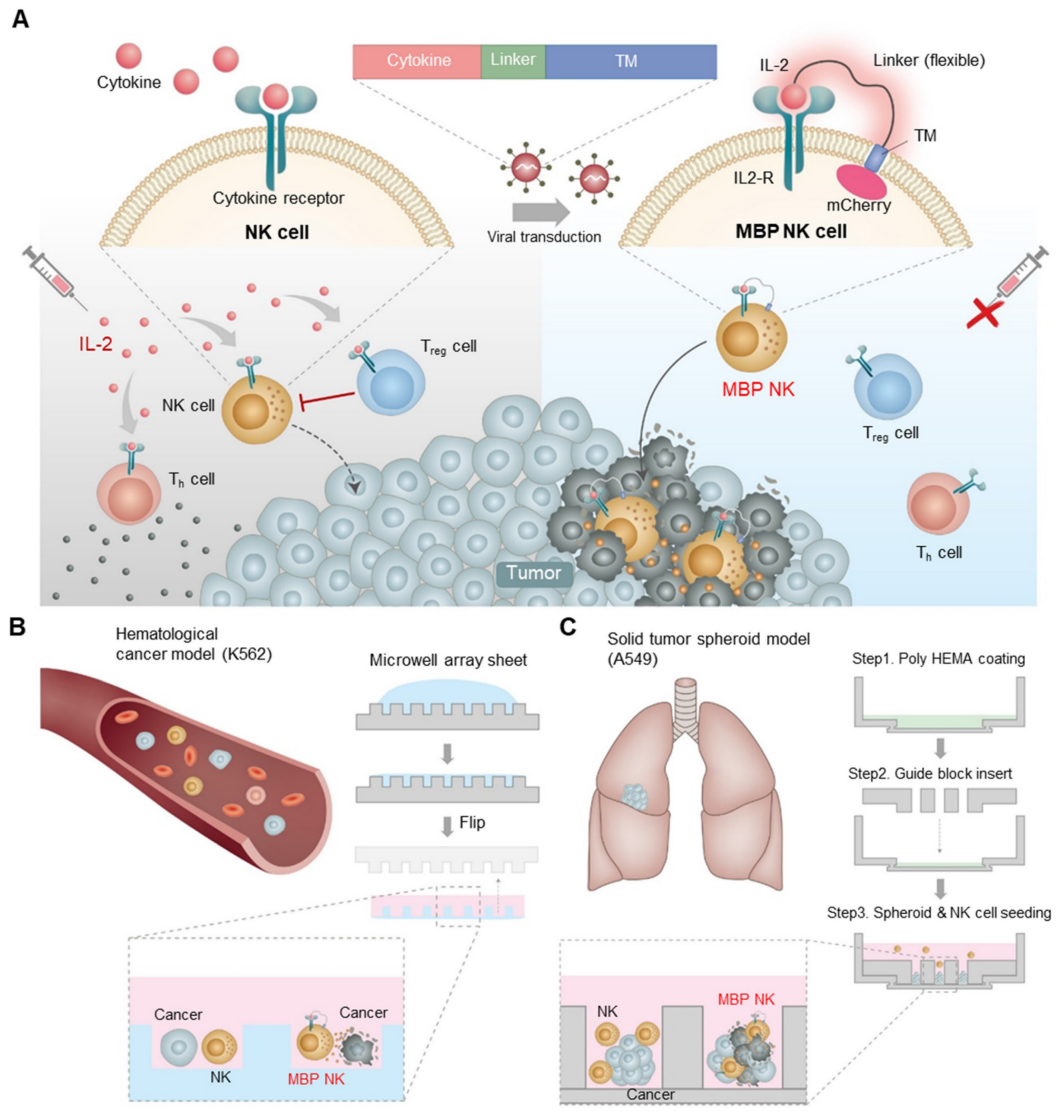
** Schematic diagram of the MBP technology in NK cells and expected role in the tumor site. (A)** Schematic illustration of cytokine engineered MBP NK cells and functional difference between parental NK-92 cells and MBP NK cells. Because of self-stimulation via MBP IL-2, MBP NK cells can survive IL-2 deficient environment as well as can reduce systemic risks of hyperimmune activation induced by exogenous treatment of IL-2. **(B)** The fabrication of the microwell array sheet to generate a hematological tumor model with K562 cancer cells. **(C)** In the solid tumor spheroid model, A549 spheroid and NK-92 cells or MBP NK cells are co-cultured and observed using confocal microscopy. The chambers are made with a guide block insert, and the bottom surface is coated with poly hydroxyethyl methacrylate (MEMA).

Here, we developed a novel membrane-bound protein (MBP) technology, which tethers IL-2 to the plasma membrane of NK-92 cells through a flexible linker (Figure 1A). We hypothesized that stable expression of MBP IL-2 (IL-2-flexible linker-transmembrane domain) on the NK-92 cell surface could induce an autocrine signal leading to continuous self-activation and this would, in turn, improves *in vivo* persistence and cytotoxic effects without stimulations of other immune cells. These self-activated NK cells are expected to have better cytotoxic effects than parental NK cells against solid tumors, wherein the IL-2 required for NK activation is deficient 11. Under the IL-2-deficient conditions of tumor microenvironment (TME), our MBP IL-2-expressing NK-92 (MBP NK) cells indeed showed improved proliferation and higher antitumor activity against a hematological tumor model and a solid tumor spheroid model compared to the parental NK-92 cells (Figure 1B and C). Interestingly, they also exhibited both faster and more infiltration into the lung tumor spheroid model, suggesting higher potency in growth inhibition of solid tumors. The MBP NK cells more effectively inhibited tumor growth in the K562 xenograft mouse model and induced more IFN-γ production without significant change in body weight. Based on this study, we expect our MBP NK to be one of the promising solutions for next generation NK cell therapy with superior clinical outcomes.

## Results and Discussion

### Generation and Characterization of MBP NK Cells

**Figure 2:**
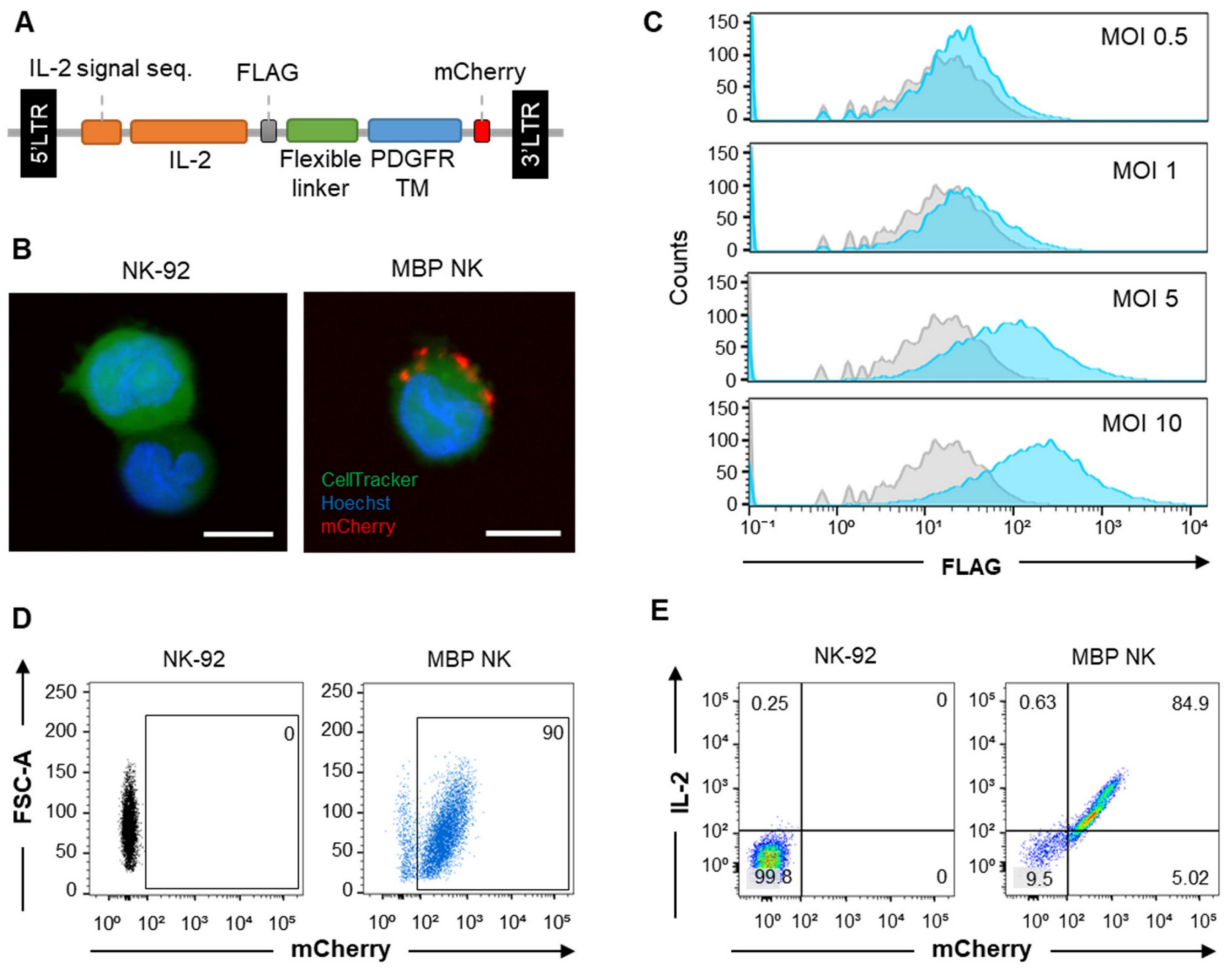
** MBP IL-2 engineering in NK-92 cells. (A)** Schematic representation of MBP IL-2 expression cassette. The ORF encoding MBP IL-2 was cloned in lentiviral vector to deliver to NK-92 cells. **(B)** Representative confocal image of parental NK-92 (left) and MBP NK (right). MBP NK cells were labeled with CellTracker (green), and nuclei were stained with Hoechst (blue). The MBP IL-2 structure was identified as red color expressing of mCherry at the C-terminal (red). Scale bar, 10 μm. **(C)** The MBP IL-2 positive cells according to different MOI analyzed by flow cytometry. After transduction, cells were labeled with PE-conjugated anti-flag tag antibody. The histogram of blue color is shown as MBP IL-2 expressing cells. The isotype control is shown as a grey color in all figures. **(D)** The expression of mCherry in NK-92 (left) and MBP NK cells (right) were analyzed by flow cytometry. **(E)** NK-92 cells and MBP NK cells were labeled PE-conjugated anti-IL-2, the expression of mCherry and IL-2 in NK-92 (left) and MBP NK cells (right) were analyzed by flow cytometry.

NK-92 cells are an IL-2-auxotrophic, natural killer cell line derived from peripheral blood mononuclear cells (PBMCs) of non-Hodgkin's lymphoma (NHL) patients 17. To generate soluble IL-2-free, self-activating NK-92 cells, we constructed a lentiviral MBP IL-2 plasmid in which IL-2 was fused to the platelet-derived growth factor receptor (PDGFR) transmembrane domain through a flexible linker and mCherry fluorescence protein at the C-terminus (Figure 2A). Membrane localization of mCherry signal from the fluorescence imaging of MBP NK cells indicated membrane expression of the MBP IL-2 construct (Figure 2B). We also performed MBP IL-2 transduction at escalating multiplicities of infection (MOIs) (Figure 2C) to maximize transduction efficiency without excessive genomic integrations. Since MOIs of 5 and 10 showed similar transduction efficiency (~25%), the NK cells transduced at MOI of 5 was selected to establish the stable cell line. Finally, maintenance of transduced MBP IL-2 was observed via fluorescence-activated cell sorting (FACS) analysis and at the fifth passage after initial sorting, the MBP NK cells showed retention of ~85% of the mCherry/IL-2 double-positive population (Figure 2D and E). In addition, we performed multiple sorting procedures on MBP IL-2 positive cells based on the intensity of mCherry signal because the transgene introduced into the NK-92 cells lacked any antibiotic selection markers, which precludes the ability to selectively proliferate cells with low levels of IL-2 expression. The MBP NK cells after the multiple sorting exhibited homogeneous membrane-bound IL-2 expression (Figure S1, Supporting Information). Moreover, when confirming the stability of membrane bound IL-2 from the MBP NK, we could not detect the shedding form of membrane bound IL-2 in the supernatant collected over time, suggesting no significant shedding of membrane bound IL-2 (Figure S2, Supporting Information). Based on these results, we successfully engineered NK-92 cells with MBP IL-2 to generate exogenous cytokine-free, self-activated NK cells (MBP NK cells).

### MBP NK Cell Proliferation and Long-term Survival without IL-2 Supplementation

**Figure 3:**
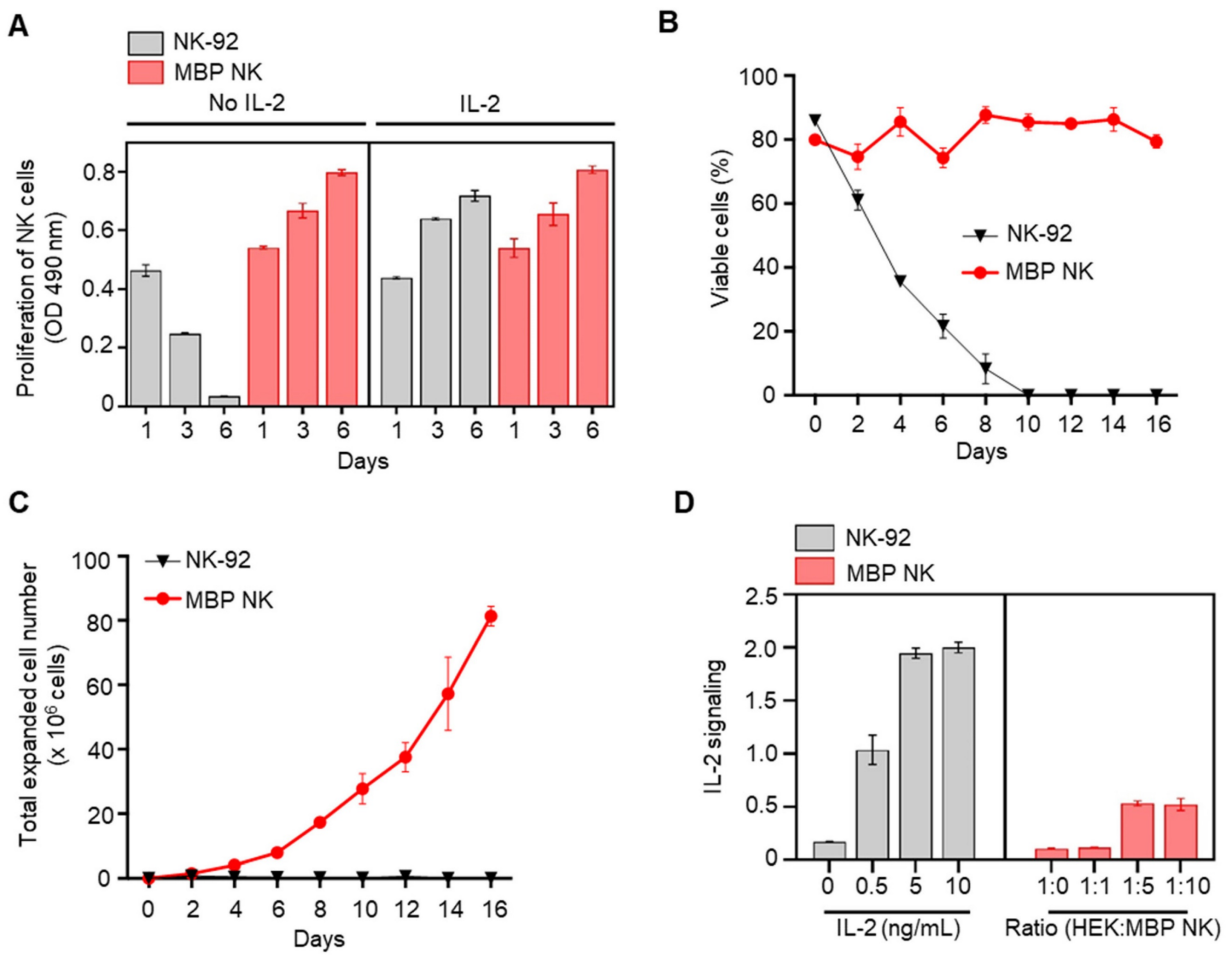
***In vitro* proliferation and expansion of NK cells by MBP IL-2 engineering and the IL-2 signal transmission in a cis-acting mechanism. (A)** Proliferation of NK-92 cells (gray color) and MBP NK cells (red color) in the presence or absence of IL-2 were investigated by an MTS assay. Means ± SDs of triplicate determinations are shown. **(B)** Viability of the parental and the MBP NK cells over time in the absence of IL-2 supplement in growth medium. The MBP NK cells were continuously maintained, with approximately 70% viability. **(C)** Expansion of parental NK-92 and MBP NK cells without exogenous IL-2 addition. While the parental NK-92 cells did not proliferate and exhibited a reduced cell number, the MBP NK cells proliferated appropriately, with an estimated doubling time of 42 hours. **(D)** Evaluation of the autocrine effect of MBP IL-2 engineering. IL-2 signal reporter HEK cells were purchased from InvivoGen (# hkb-il2) to monitor the activation of the JAK-STAT pathway induced by IL-2. The level of secreted embryonic alkaline phosphatase (SEAP) from reporter HEK cells did not increase proportionally even when the proportion of cocultured MBP NK cells was increased, which confirmed that the MBP IL-2 on the MBP NK cells mainly acts in a cis manner dominantly.

Since IL-15 and -21 are also known to stimulate the proliferation of NK cells like IL-2 18, we also compared the distribution of these cytokine receptors on the surface of NK-92 cells to identify the suitable cytokine for NK-92 engineering. The IL-2 specific receptors such as CD122 (IL-2Rβ) and CD132 (IL-2Rγ) were expressed at the highest level compared to IL-15 or -21 specific receptors suggesting that IL-2 is a major cytokine responsible for sustaining NK-92 cell survival (Figure S3, Supporting Information). Next, to confirm whether MBP NK cells can proliferate without exogenous IL-2, we compared the viability between MBP NK cells and parental NK-92 cells with or without IL-2 treatment by MTS assay. In groups treated with IL-2, both groups of NK cells showed a similar proliferation rate. In groups without IL-2, however, the proliferation of parental NK-92 cells was significantly reduced, while the proliferation of MBP NK cells remained unchanged compared to the groups treated with IL-2. Interestingly, additional IL-2 treatment did not enhance MBP NK cell proliferation, implying that MBP IL-2 enabled a self-sufficient proliferation of the MBP NK cells (Figure 3A). We then compared the expansion rate between MBP NK cells and parental NK-92 cells without IL-2. The cellular growth and viability of the MBP NK cells were stably sustained for more than 2 weeks without IL-2. Over 16 days, the MBP NK cells showed an approximately 32-fold increase in the total cell count, whereas parental NK-92 cells did not survive (Figure 3B and C). The NK-92 cell line was established from peripheral blood mononuclear cells collected from a male patient, aged 50 years, diagnosed with non-Hodgkin's lymphoma. While the cells exhibit immortality *in vitro* with IL-2 supplementation, the MBP NK cells displayed continuous growth in *vitro in* the absence of IL-2, which might raise safety concerns. The survival of MBP NK cells *in vivo* is subject to the intricacies of the human body, which deviate from their behavior *in vitro*. These complexities result from anti-inflammatory cytokines released by other regulatory immune cells or direct interactions with Treg cells in environments such as inflammatory sites or tumor microenvironments, even in the presence of IL-2. To minimize the concerns of the safety issue, the implementation of an inducible caspase-9 suicide gene system as a "safety switch" could be considered 19. Additionally, pretreatment with gamma ray irradiation to prevent uncontrolled growth may also be a practical solution for use in clinical trials 20.

Finally, we investigated whether MBP IL-2 transactivates neighboring cells or acts only via a cis-acting manner since the trans-presentation of IL-2 mediates several immunological processes, including DC-mediated T cell activation and cluster-dependent NK cell activation 21, 22. The IL-2 reporter assay showed that MBP NK cells could trans-present MBP IL-2 to neighboring IL-2 reporter cells. However, the extent of trans-activation was only approximately 25% of the cis-activation, as determined by direct IL-2 treatment (Figure 3D) and in the MBP NK cell and parental NK-92 cell coculture experiments, the MBP NK cells were predominantly expanded (~80%) after 8 days, suggesting that the MBP NK cells predominantly utilize cis-presentation of MBP IL-2 (Figure S4, Supporting Information). Moreover, we determined the distribution and long-term persistence of MBP NK *in vivo*. To measure the persistence of MBP NK, we infused 5 × 10^6^ DIR 750 labeled NK-92 or MBP NK cells per mouse via the tail vein. Additionally, recombinant human IL-2 was only administered to the mice infused with NK-92, delivered via an intraperitoneal method. Using the IVIS Spectrum instrument (PerkinElmer, Waltham, MA, USA), we measured the radiance of the DIR 750 labeled NK-92 or MBP NK cells at specific time points (0, 40 min, 2 h, Day 1, Day 3, Day 5, and Day 10). After 24 hours, the total fluorescent intensity of the mice in the MBP NK-infused group was slightly higher than that of the NK-92 group. Furthermore, compared to NK-92, MBP NK showed greater infiltration into the lymph nodes and femur (*see the red circles* in Figure S5, Supporting Information). This preferential migration of MBP NK into the lymphoid organs, potentially triggered by the expression of cognate chemokine receptors such as CXCR4 or CXCR7 following stimulation with membrane-bound IL-2, may demonstrate its potential to activate anti-tumor response-related immune cells. At 14 days post-transplantation, the mice were sacrificed and the presence of MBP NK was determined by determining the fluorescence intensity of the organ-resident NK-92 or MBP NK (Figure S6, Supporting Information). As anticipated, MBP NK showed a higher level of fluorescence compared to that of NK-92 and the control group. To conclude, these results suggest that the membrane-bound IL-2 approach can promote the proliferation and persistence of the NK cells *in viv*o, which could overcome the current limitations of soluble IL-2-dependent NK cell therapy.

### MBP NK Cells Exhibit Enhanced Tumor Killing by Strong Secretion of Lytic Granules

**Figure 4:**
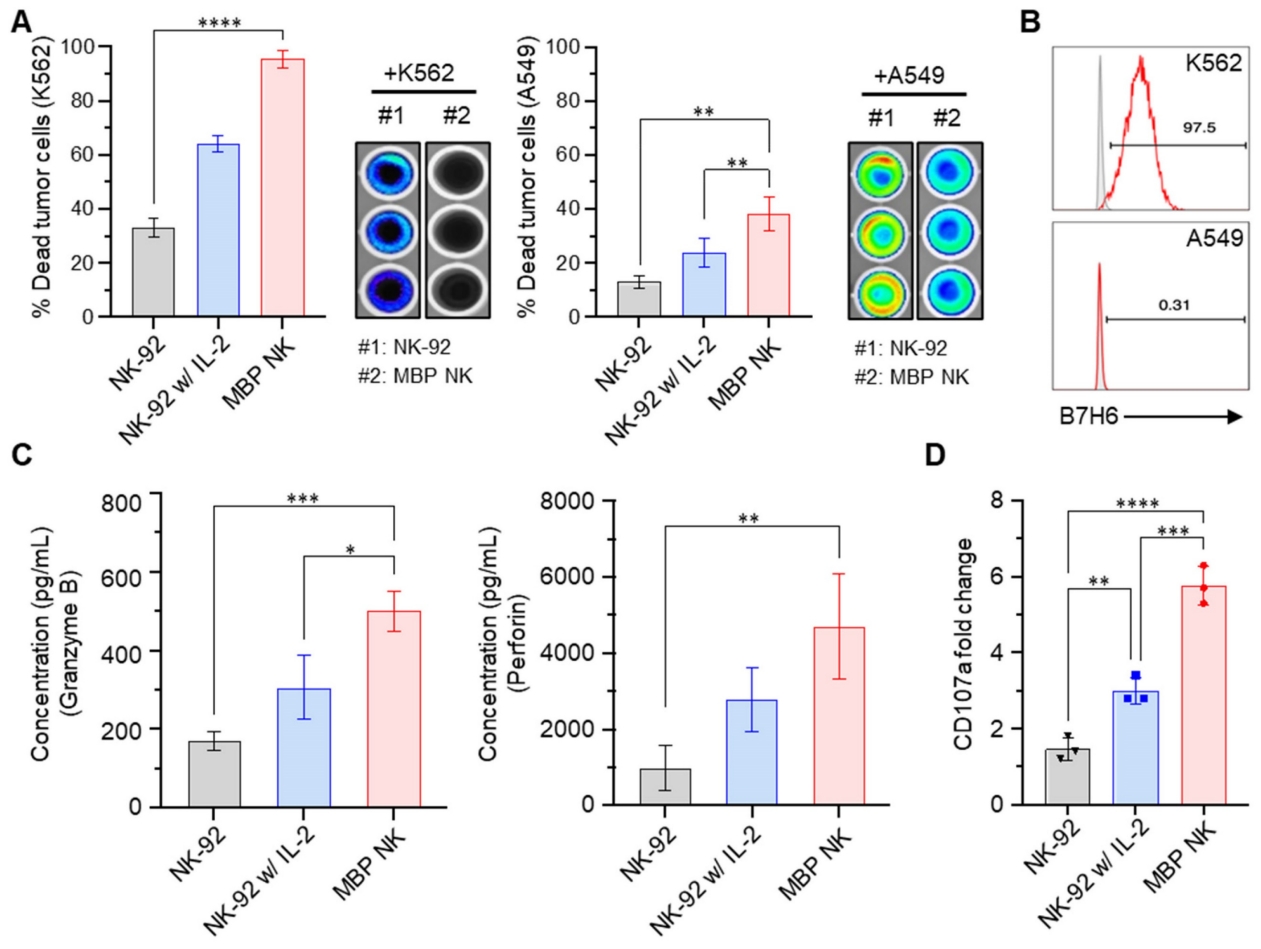
** MBP NK cells exhibit enhanced anti-tumor effects compared to parental NK cells. (A)** MBP NK mediated cancer cell killing. K562 (left) and A549 cells (right) that stably expressed luciferase were co-cultured with NK-92 or MBP NK cells at an E:T ratio of 5:1. The percentage of dead tumor cells was determined by quantifying bioluminescence. The statistical significance was determined by one-way ANOVA with Tukey's multiple comparison test. *, p = 0.0280, **, p = 0.0018; ****, p < 0.0001. **(B)** The expression level of B7H6 on the surface of K562 (top) and A549 cells (bottom), which is one of the major activating immune ligands on cancer cells. NKp30 expressed by NK cells can recognize and kill B7H6 expressing tumor cells. **(C)** The quantitative measurement of lytic granules released from NK-92 and MBP NK cells by ELISA. The concentrations of secreted granzyme B (left) and perforin (right) from NK-92 cells (absence or presence of IL-2) and MBP NK cells were measured in the supernatant from the co-culture with K562 cells. The statistical significance was determined by ordinary one-way ANOVA with Tukey's multiple comparison test. *, p = 0.0138; **, p = 0.0091; ***, p = 0.001. **(D)** Fold change in CD107a expression analyzed by flow cytometry. The plot was generated by Prism 8 software. The statistical significance was determined by ordinary one-way ANOVA with Tukey's multiple comparison test. **, p = 0.0075; ***, p = 0.0003; ****, p < 0.0001. From all the analyses, statistically insignificant (ns) results are not shown.

**Figure 5:**
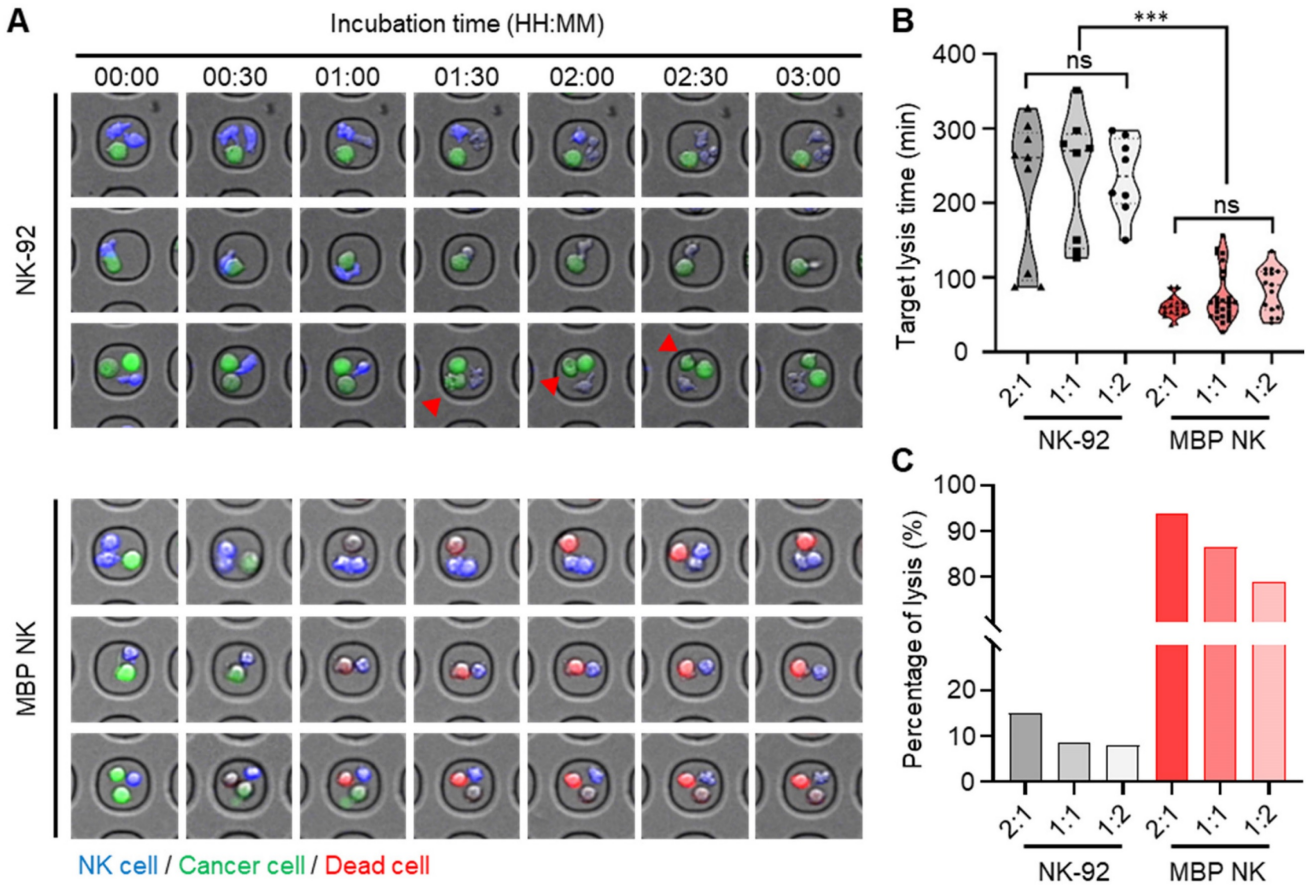
** Quantitative analysis of cytotoxicity mediated by parental NK and MBP NK cells at the single-cell level in hematological cancer cells (K562). (A)** Representative time-lapse images of the NK cells (top) and the MBP NK cells (bottom) cocultured with K562 cells were obtained with a confocal microscope. Both types of NK cells were stained with CellTracker^TM^ blue, and K562 cells were labeled with calcein AM. Compared to the parental NK cells, the MBP NK cells killed the myelogenous leukemia cells very rapidly at all E:T ratios. The red arrows indicate that the hematological cancer cells were still alive, although the NK cells were actively interacted with the cancer cells and seemed to destroy their membranes. **(B)** Comparison of target lysis time between the NK and MBP NK cells at different E:T ratios. The time was recorded when the first target cell died. The MBP NK cells killed the target cells more efficiently, with less variation, and the E:T ratio did not affect the time required for target lysis. **(C)** Probability of target cell lysis by the NK and MBP NK cells at different E:T ratios. Compared to the parental NK cells, the MBP NK cells showed superior lysis performance, and the E:T ratio affected lysis performance. Lysis was evaluated by analyzing 319 microwell chambers. The statistical analysis was performed with an unpaired two-tailed t-test, ***, ρ < 0.001; ns = not significant.

Sustaining both proliferation and cytotoxicity *in vivo* is essential for next-generation NK cell-based therapy. Since MBP NK cells possess improved proliferation capacity due to membrane-bound IL-2, we next compared their tumor killing activity with NK cells or NK cells treated with soluble IL-2 by co-culturing with two different cancer cell lines, K562 and A549 that stably express luciferase. Quantification of dead cells by bioluminescence indicated enhanced tumor killing ability of MBP NK cells against both K562 and A549 cells compared to parental NK cells (Figure 4A). Interestingly, A549 cells were more resistant to killing by NK cells compared to K562 and this result may have arisen from the minimal expression of B7-H6 in A549, which is the ligand for Nkp30, one of the important activating receptors of NK cells (Figure 4B). We next measured the level of granzyme B and perforin secretion as NK cells are known to utilize the cytolytic granule secretory pathway to kill abnormal cells such as cancer cells 23. Intriguingly, the MBP NK cells showed a more than 2-fold increase in granzyme B secretion and an approximately 3-fold increase in perforin secretion in response to co-cultured K562 target cells compared to the parental NK-92 cells (Figure 4C). Surface expression of the lysosomal protein CD107a, which is located on the cell membrane during the NK cell degranulation process, was also significantly increased in MBP NK cells (Figure 4D). To analytically investigate *in vitro* antitumor efficacy of the MBP NK cells, we designed microwell chamber arrays that allow single-cell imaging of the real-time behavior of NK cells in response to target cancer cells such as K562 (Figure 5A and Videos S1-S6, Supporting Information). Under IL-2 deficient conditions, all effector-to-target (E:T) ratios resulted in MBP NK cells performing faster and stronger tumor killing activities compared to parental NK-92 cells. Target killing started 230 minutes (average) after co-incubation for the parental NK-92 cells while ethidium homodimer 1 (red)-positive dead cancer cells were found less than one hour for MBP NK cells. (Figure 5B). It was noted that while the target lysis time was highly variable among individual parental NK cells, the MBP NK cells showed a much faster and more consistent tumor killing speed. Moreover, the analysis of 319 microwells showed 93.8%, 86.6%, and 78.9% target cell lysis by MBP NK cells at ET ratios of 2:1, 1:1, and 1:2, respectively, while the parental NK-92 cells showed only 15%, 8.7%, and 7.9% cytolysis under the same ET ratios (Figure 5C). These results indicate that our MBP NK cells are also armed with stronger anti-tumor activity with the elevated secretion of lytic granules.

### Infiltration and Cytotoxicity of MBP NK Cells in a Spheroid Tumor Model

Although several NK cell-based therapies have shown clinical benefits, particularly against hematological malignancies, most solid tumors remain impregnable due to the limited infiltration of NK cells into solid tumors 24. The avascular and hypoxic microenvironment of solid tumors induced by chronic inflammation directly suppresses the infiltration of NK cells and the IL-2-deficient environment cannot promote survival and proliferation of the infiltrated NK cells, leading to a poor antitumor response. In addition, a recent clinical study suggested that the infiltrated NK cell population itself is an important sign of improved clinical outcomes 25. In this context, we hypothesized that self-activating MBP NK cells would demonstrate improved survival and cytolytic activities after tumor infiltration, as opposed to conventional NK cell-based therapies that rely on exogenous IL-2 for survival and cytolytic activities.

**Figure 6:**
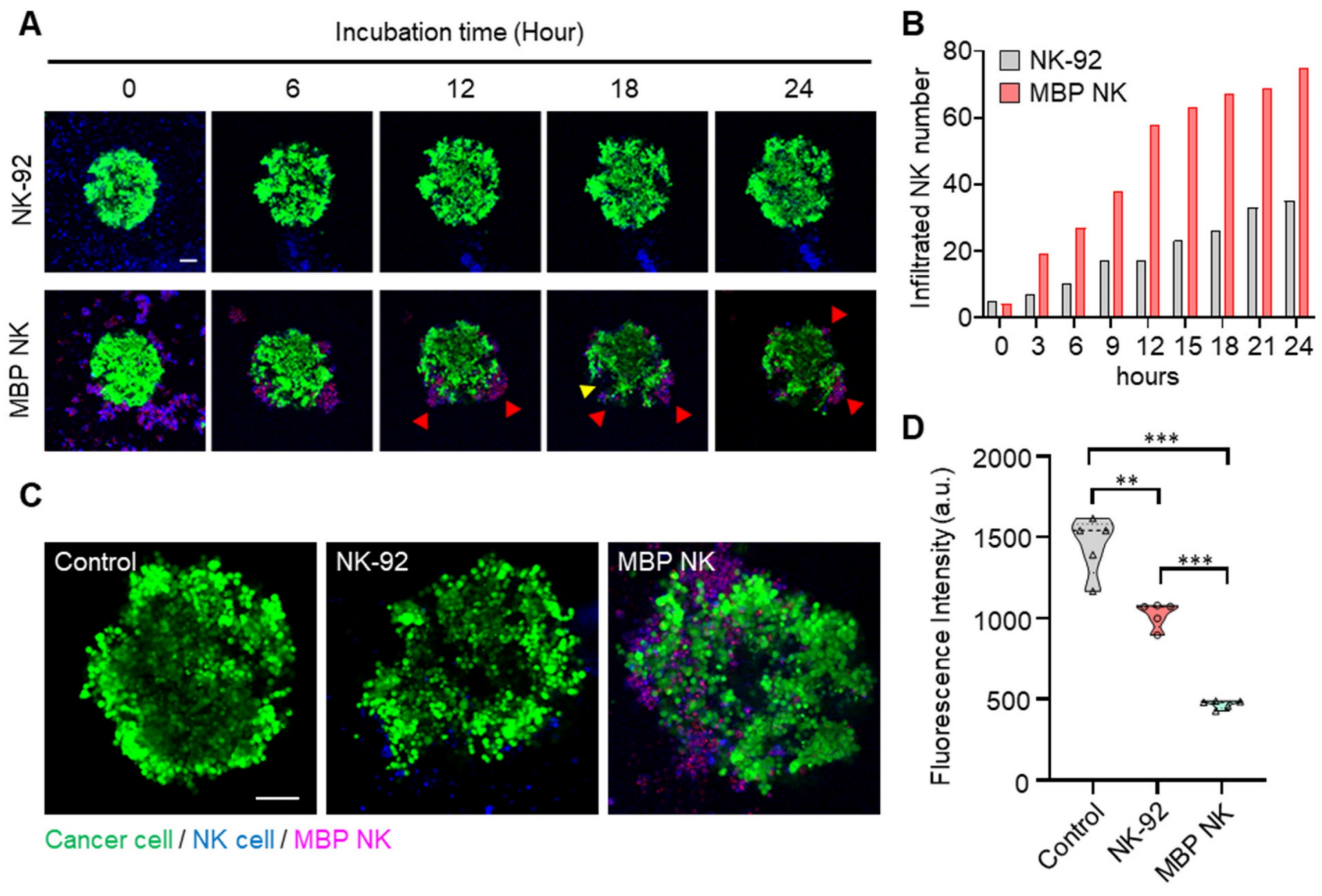
** The infiltration and cytolysis abilities of the MBP NK cells in the lung tumor spheroid model. (A)** Time-lapse confocal images of A549 tumor spheroids stained with calcein AM and cocultured with NK cells or MBP NK cells. Red arrows indicate clusters of MBP NK cells around the tumor spheroid. The cluster of MBP NK cells exhibited effective cytolysis, creating a massive dead zone in the tumor spheroid (*see the yellow arrow*). **(B)** Number of infiltrating cells for the NK and MBP NK cells after co-cultivation with A549 spheroids for 24 hours. The number of infiltrating cells was dramatically increased in the MBP NK cells compared to the parental NK cells. **(C)** Representative confocal images of A549 spheroids alone and spheroids co-cultured with NK cells or MBP NK cells after 48 hours. Scale bar, 100 μm. The MBP NK cells showed more infiltration inside the tumor spheroid. **(D)** Quantitative analysis of live cancer cells. The cancer spheroids presented significantly weaker fluorescent signals when cocultured with the MBP NK cells. Fluorescence was analyzed by reviewing the confocal images captured after 48 hours of cocultivation (n=5). The statistical analysis was performed with an unpaired two-tailed t-test, **, ρ < 0.01; ***, ρ < 0.001.

To confirm this hypothesis, we reconstituted an A549 spheroid tumor model mimicking the avascular hypoxic tumor microenvironment. Over 24 hours of A549 spheroid tumor and NK cell coculture, we monitored the migration and infiltration of the parental NK and MBP NK cells into the A549 spheroid model (Videos S7 and S8, Supporting Information). MBP NK cells tended to immediately cluster with neighboring cells, and the clustered MBP NK cells were attached to the lung tumor spheroid. This phenomenon seems to be beneficial for antitumor activity because the clustered MBP NK cells would create a locally high E:T ratio near the cancer cells and increase cytolytic ability, which was consistent with the previous results. As shown in Figure 6A, the cluster of MBP NK cells exhibited effective cytolysis, creating a massive dead zone (*see the yellow arrow*). Overall, the MBP NK cells showed significant and vigorous infiltration capability compared to that of parental NK cells, and the number of infiltrated MBP NK cells was over 2-fold higher than that of the parental NK cells at every time point (Figure 6A and B). This corresponded to the cytolytic activity inside the tumor spheroid. As shown in Figure 6C and D, the MBP NK cells not only severely destroyed the tumor spheroid integrity and structure but also substantially lysed the target spheroid. Taken together, these data demonstrate the superior infiltration capability and cytolytic activity of MBP NK cells.

### Cytotoxicity of MBP NK Cells in a Mouse Xenograft Model

**Figure 7:**
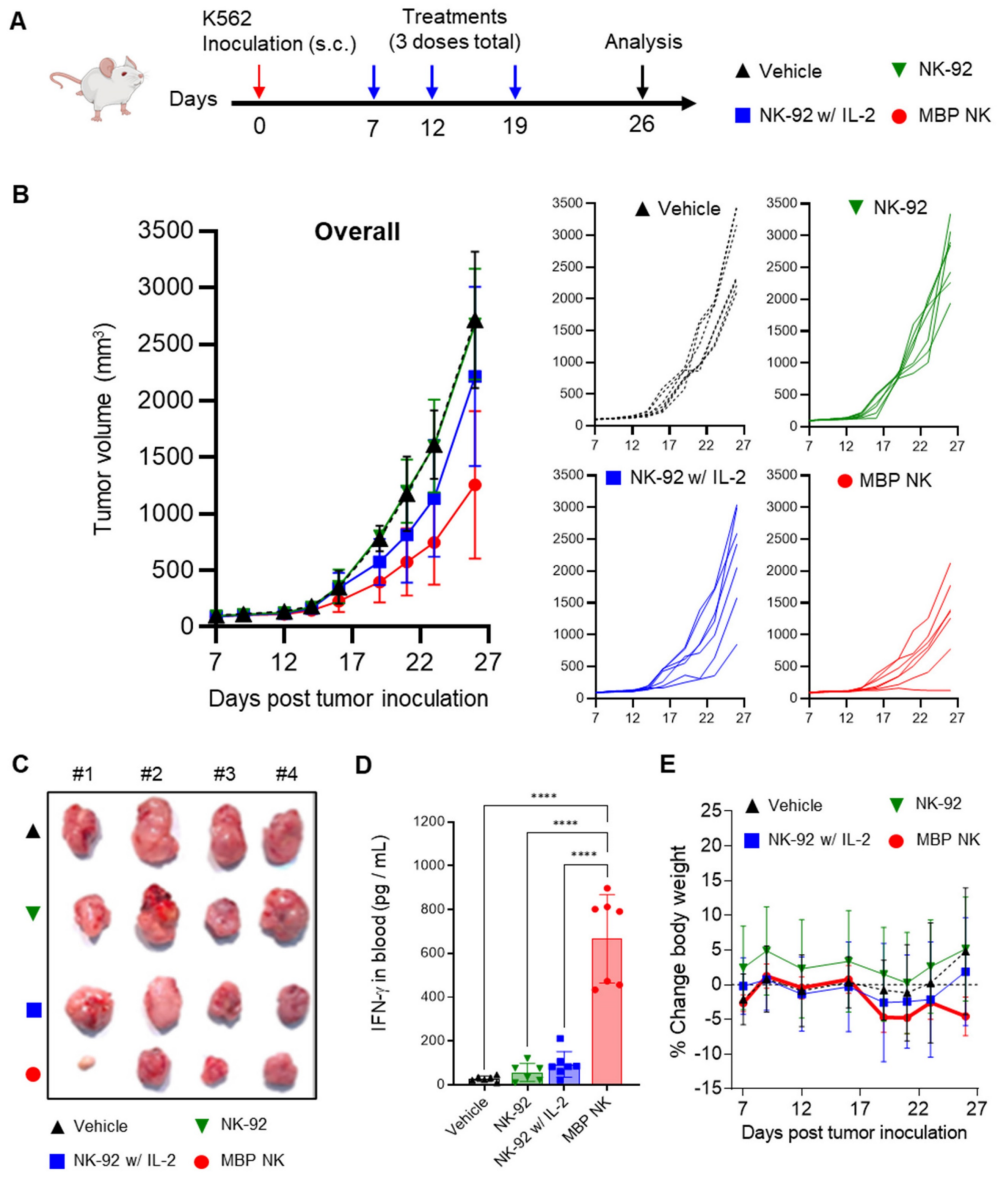
***In vivo* anti-tumor efficacy of MBP NK in a solidified K562 tumor mouse model. (A)** Experimental scheme of K562 xenograft mouse model. Female NIG mice, aged 7-8 weeks (n = 7 / group) were inoculated subcutaneously in their right flank with K562 tumor cells. When tumor volumes reached ~50 mm^3^, after 7 days of tumor inoculation, mice were intravenously administered MBP NK or NK-92 at a dose of 5 × 10^6^ cells / mouse (3 doses total). **(B)** Tumor volume analysis to quantitatively determine tumor growth inhibition. Overall and individual responses are shown. Treatment of MBP NK resulted in potent tumor growth inhibition compared to NK-92 or IL-2 co-administrated NK-92. **(C)** A picture of tumors from each mouse after study termination. **(D)** On day 26, blood samples were collected from each mouse and IFN-γ level was analyzed by ELISA. **(E)** Body weight changes (%) in mice after injection of the indicated NK cells. All groups showed body weight change within the 5% range. The statistical significance was determined by ordinary one-way ANOVA with Holm-Šidák's multiple comparison test, ****, p < 0.0001. From all the analyses, statistically insignificant (ns) results are not shown.

To evaluate the anti-tumor efficacy of the MBP NK *in vivo*, we first established a xenograft mouse model by subcutaneous (s.c.) injection of the K562 cell line into NOD.CB17/Prkdcscid/JKrb/ IL2 receptor γ-/-(NIG) mice mimicking the solid tumor. This model was chosen as the absence of lymphocytes in this model would create IL-2 deficiency and therefore, allow the assessment of *in vivo* anti-tumor efficacy of MBP NK compared to parental NK-92 cells. Four different injections - vehicle, NK-92 only, NK-92 with recombinant human IL-2 (rhIL-2) or MBP NK cells were given starting on 7 days after inoculation of the K562 cell line once a week for a total of three doses (Figure 7A). The tumor growth inhibition was evaluated by quantification of tumor volume, plasma concentration of human interferon-γ (rhIFN-γ) and body weight. As a result, tumors were most undergrown in the mice injected with MBP NK compared to other groups (Figure 7B and C). The concentration of plasma rhIFN-γ measured after the study termination was also highest in the MBP NK group (Figure 7D) and this indicates the efficiency of MBP IL-2 in eliciting an immune response of NK cells. The body weight change of mice from all the groups was within the 5% range (Figure 7E), meaning that no significant metabolic side-effects were observed in this study. From these results, the MBP NK cells demonstrated superior tumor growth inhibition compared to NK-92 only or NK-92 with the rhIL-2 group, emphasizing greater potency generated by MBP IL-2. Interestingly, in one of the seven cases, MBP NK nearly completely suppressed the growth of the K562 tumor in the mouse model. Despite this noticeable result, it is believed that there are limitations in mouse tumor model and the MBP NK itself that need to show more excellent anti-tumor efficacy. The tumor growth inhibition observed in Figure 7B was solely a result of the anti-tumor activity of MBP NK. It is hypothesized that if a more complete immune cells were present in the mouse model, such as T cells or NK cells, communication with activated MBP NK would result in an even stronger inhibition of tumor growth. Upon recognizing tumor cells through binding between NCRs and its cognate antigens, MBP NK transfers stimulants such as IFN-γ to adjacent immune cells, thereby triggering an immune response. Therefore, we believe that the full potential of MBP NK would be realized in an immune-competent host rather than an immune-deficient one. Furthermore, to counteract the mechanisms by which cancer cells evade immune surveillance, combining multiple cancer targeting strategies, such as the utilization of a chimeric antigen receptor (CAR) in conjunction with MBP, may lead to improved and sustained anti-tumor efficacy in clinical settings.

## Conclusion

NK cell therapy has received high attention as a novel immunotherapy agent that can ensure both potency and safety. However, the therapeutic potential of NK cells has not been fully accomplished because of NK cells' high dependence on cytokine for proliferation and cytolytic activity. Results from combination therapy with cytokines such as IL-2 were not encouraging since only a small portion of IL-2 could reach NK cells due to the presence of IL-2 receptors across multiple cell types including regulatory T cells that downregulate the activity of NK cells 14. More potent anti-tumor activity of MBP NK compared to parental NK-92 with soluble IL-2 during *in vivo* experiment also suggests the importance of efficient and accurate delivery of cytokines in realizing the therapeutic potential of NK cells. Safety concerns such as capillary leakage syndrome 15 and influence on immune reconstitution after lymphodepletion during treatment of hematological cancer patients are additional shortcomings of IL-2 treatment 26. In other words, while there is efficacy-safety trade-off in IL-2 co-treatment during NK cell therapy, our MBP approach is a novel strategy that can overcome such limitation. With enhanced antitumor activity under cytokine deficient conditions *in vivo*, strong secretion of lytic granules and superior tumor infiltration capacity, MBP NK is expected to achieve high therapeutic index in clinical application. Finally, we anticipate that the additional introduction of other functional membrane proteins such as chimeric antigen receptor in this MBP NK platform could further expand the therapeutic benefits of NK cells.

## Methods

### Cell Lines and Cell Culture

The human cell line NK-92 (NK cell lymphoma) was purchased from Korean Collection for Type Cultures (KCTC, Daejeon, Korea). The cell lines K562 (human chronic myelogenous leukemia, CML) and A549 (human lung adenocarcinoma) were purchased from Korean Cell Line Bank (KCLB, Seoul, Korea). Luciferase-tagged K562 cells (K562-luc) were purchased from American Type Culture Collection (ATCC, Manassas, VA, USA), and luciferase-tagged A549 cells (A549-red-fluc) were kindly provided by Prof. Kyungmoo Yea (DGIST, Daegu, Korea). NK-92 cells were maintained in Minimum Essential Medium (MEM) α with nucleosides (Thermo Fisher Scientific, Rockford, IL, USA) supplemented with 0.02 mM folic acid (Sigma-Aldrich, St. Louis, MO, USA), 0.2 mM myoinositol (Sigma-Aldrich), 0.1 mM 2-mercaptoethanol (Thermo Fisher Scientific), 12.5% fetal bovine serum (FBS; Thermo Fisher Scientific), 12.5% horse serum (Thermo Fisher Scientific), penicillin-streptomycin (Thermo Fisher Scientific) and 100 IU/mL recombinant human IL-2 (Corning, Corning, NY, USA). MBP NK cells were cultured in MEM α with nucleosides (Thermo Fisher Scientific) supplemented with 0.02 mM folic acid (Sigma-Aldrich), 0.2 mM myoinositol (Sigma-Aldrich), 0.1 mM 2-mercaptoethanol (Thermo Fisher Scientific), 12.5% FBS (Thermo Fisher Scientific), 12.5% horse serum (Thermo Fisher Scientific), and penicillin-streptomycin (Thermo Fisher Scientific). The cells were incubated at 37 °C in a 5% CO_2_ atmosphere. A549 and A549-red-fluc cells were routinely cultured in PRMI1640 (Welgene, Gyeongsan, Korea) supplemented with 10% fetal bovine serum (Thermo Fisher Scientific) and antibiotics-antimycotics (HyClone Laboratories, Inc., South Logan, UT, USA). K562 and K562-luc cells were cultured in Improved Minimum Essential Medium (IMEM; Thermo Fisher Scientific) with 10% fetal bovine serum (Thermo Fisher Scientific) and antibiotics-antimycotics (HyClone Laboratories, Inc.). A549 spheroids were produced by seeding 5,000 cells per well on ultralow-attachment round-bottom 96-well plates and maintained under the same conditions as the A549 cells.

### Production of MBP NK Cells

To produce lentiviruses, 293 FT cells were purchased from Thermo Fisher Scientific and maintained according to the manufacturer's instructions. We produced lentiviruses that could express IL-2 in the membrane-bound form. Lentiviral supernatants were added to NK-92 cells with 8 μg/mL polybrene (Sigma-Aldrich), centrifuged for 90 minutes at 1200 ×g and then incubated in a CO_2_ incubator for 24 hours. The virus-containing medium was replaced with a fresh growth medium. After 4 days, MBP NK cells, which were mCherry positive, were sorted using a FACS flow cytometry system (BD Biosciences, Franklin Lakes, NJ, USA).

### Imaging of MBP NK Cells

For ease of visualization, the cytoplasm and nucleus of sorted MBP NK cells were labeled with Celltracker^TM^ green (Invitrogen, Carlsbad, CA, USA) and Hoechst fluorescent dye (Invitrogen), respectively. After removing the culture medium, the fluorescent dye was injected into a serum-free medium, and the cells were incubated in a CO_2_ incubator for 30 minutes. Fluorescence images were acquired using a Nikon confocal microscope.

### Identification of MBP NK Cells

To evaluate the efficiency of viral transduction, MBP NK cells were labeled with a PE-conjugated anti-DYKDDDDK tag antibody (FLAG tag; Biolegend, San Diego, CA, USA) and analyzed using a FACS flow cytometry system (BD Biosciences). The population of cultured MBP NK cells was analyzed by observing the ratio of mCherry-positive cells using a FACS flow cytometry system (BD Biosciences). To confirm the expression of MBP IL-2, NK-92 cells and MBP NK cells were washed twice with phosphate-buffered saline (PBS; Welgene) and stained with a PE-conjugated anti-IL-2 antibody (R&D Systems, Minneapolis, MN, USA) in staining buffer (BioRad, Hercules, CA, USA) for 30 minutes at 4 ℃. The population of MBP NK cells was visualized based on the obtained flow cytometry data using FlowJo 10.8.0 software (FlowJo LLC, Ashland, OR, USA).

### Proliferation Assay

To compare the proliferation ability of MBP NK cells and NK-92 cells, the total number of live cells was counted, excluding cells stained with trypan blue (Thermo Fisher Scientific), among 10,000 cells per well seeded in 96-well plates. After one or three days, 200 μL of MTS dye solution (Promega, Madison, WI, USA) was added to each well and incubated for 4 hours. Optical density values at 490 nm were obtained using a spectrophotometer (Molecular Devices, Sunnyvale, CA, USA), and the results were analyzed using GraphPad Prism 8 software (GraphPad Software, Inc., San Diego, CA, USA). NK-92 cells, the control used in all experiments, were then cultured in a growth medium without IL-2 for two days for accurate evaluation in a medium without IL-2 22.

### IL-2 Signaling Assay

To confirm the effect of IL-2 in engineered cells, reporter cells were used. IL-2 reporter HEK 293 cells were purchased from Invitrogen. The cells were cultured in Dulbecco's modified Eagle's medium (DMEM; Welgene) with 1% penicillin/streptomycin (Thermo Fisher Scientific). For the signaling assay, IL-2 reporter HEK cells were seeded into 96-well plates at 50,000 cells per well, and IL-2 was added at concentrations of 0, 0.5, 5 and 10 ng/mL. MBP NK cells were seeded into 96-well plates in which reporter cells were injected so that the ratio of reporter cells to MBP NK cells was 1:0, 1:1, 1:5 or 1:10. After incubation for 24 hours, 180 μL of QUANTI-Blue^TM^ Solution (InvivoGen, San Diego, CA, USA) was dispensed into each well in 96-well plates, and 20 μL of supernatant was added to the cocultured samples. Then, the 96-well plates were incubated for 1 hour. Secreted embryonic alkaline phosphatase (SEAP) levels were analyzed by spectrophotometry (Thermo Fisher Scientific) at 640 nm.

### NK Cell-released Lytic Granules

Granzyme B and perforin secreted from NK cells were investigated under coculture conditions with K562 tumor cells. NK cells were seeded at 40,000 cells per well and cocultured with 4,000 K562 cells per well for 3 hours. The concentrations of lytic granules were determined using a granzyme B ELISA kit (Mabtech, Cincinnati, OH, USA) and a human perforin ELISA kit (Abcam, Cambridge, MA, USA). After the experiments were performed according to the manufacturer's instructions, the results were read using a spectrophotometer (Molecular Devices), and the concentrations of granules were analyzed using GraphPad Prism 8 software.

### Microwell Array Fabrication and Live Cell Imaging

To fabricate microwell arrays for real-time imaging, standard soft lithography methods were used. Briefly, a master mold was fabricated through a photolithography process using SU-8 negative photoresist. PDMS base was mixed with a cross-linker at a 10:1 ratio, poured onto the master mold and spin coated 500 ×g for 30 seconds so that the thickness of PDMS was 200 μm. The PDMS prepolymer was cured at 65 °C for 1 hour, and then the cured PDMS microwell array was peeled off from the master mold. The microwells were gently washed with PBS and attached to confocal dishes. To observe the cytotoxicity of NK cells against tumor cells in the microwells, NK-92 cells and MBP NK cells were stained with CellTracker^TM^ blue (Invitrogen), and K562 cells were stained with calcein AM (Invitrogen) for 30 minutes. NK-92 cells or MBP NK cells (10,000 each) were loaded into the microwell platform along with K562 cells. The coculture medium was a 1:1 mixture of NK cell growth medium without IL-2 and K562 cell growth medium. Then, ethidium homodimer 1 (Invitrogen) was added to visualize dead cells. To promote cell settling, the microwell array was centrifuged at 300 ×g for 3 minutes. Live cell imaging was performed with a Nikon confocal live cell imaging system. The probability of target cell lysis was calculated as follows: % percentage of lysis = 100 × (among the microwells that contain both NK cells and K562 cells, number of the wells that K562 showed dead signal)/(number of the microwells that contain both NK cells and K562 cells).

### Flow Cytometry

To analyze the surface markers of cells, the cells were washed twice with cell staining buffer (BioLegend) by centrifugation for 5 minutes at 300 ×g. The washed cells were blocked with Human TruStain FcX^TM^ (BioLegend) for 20 minutes at 4 °C in the dark to minimize nonspecific binding through the Fc receptor of staining antibodies. For staining of cell-surface markers, the cells were then incubated with fluorophore-conjugated primary antibodies or isotype controls in cell staining buffer for 20 minutes at 4 °C in the dark, washed with cell staining buffer and analyzed using a BD FACSVerse flow cytometer (BD Biosciences). Flow cytometric data were analyzed using FlowJo software version 10.8.0. The antibodies are described in Supporting Information Table S1.

### *In Vitro* Cytotoxicity Assays

The cytolytic activity of MBP NK cells against cancer cells was determined using bioluminescence imaging. A549 red-fluc and K562-luc cells were harvested from culture flasks, and 10,000 cells per well were seeded in white 96-well plates. After 24 hours, the number of A549 red-fluc and K562-luc cells was counted. Based on the count of cell numbers, an adequate number of NK-92 cells or MBP NK cells were added to cancer cell-seeded wells. Then, the cells were cocultured for 24 hours. After 24 hours, D-luciferin was added to the wells to a final concentration of 150 μg/mL. Bioluminescence imaging (BLI) was performed with an IVIS Spectrum instrument (PerkinElmer) on the basis of the average radiance (photons/second/cm^2^/steradian). Cancer cells incubated without effector cells were used to measure spontaneous death (reported as RLUs) and were treated with 4% paraformaldehyde for maximal killing. Triplicate wells were averaged, and the percentage of specific lysis was calculated as follows: % specific lysis = 100 × [(experimental data - spontaneous cell death)/(maximum cell death - spontaneous cell death)]. Quantitative results were analyzed using GraphPad Prism 8 software.

### Live Imaging of Spheroids

To prevent movement and attachment of spheroids during live imaging, guide blocks and surface coatings were applied to the confocal dishes. Two hundred microliters of 12% 2-hydroxyethyl methacrylate (poly HEMA; Sigma-Aldrich) solution in 95% ethanol was poured into a confocal dish and air dried overnight. The guide blocks to separate the space with an appropriate size were made of PDMS. Three milliliters of PDMS prepolymer was poured onto a confocal dish and cured at 65 °C for 2 hours. The PDMS guide blocks were peeled from the confocal dish, and small holes were punched to place the spheroids. Then, the guide blocks were attached to poly HEMA-coated confocal dishes. Celltracker^TM^ blue-stained NK-92 cells or MBP NK cells and calcein AM-stained A549 spheroids were injected into a guide block hole at an E:T ratio of 10:1. Live cell imaging was performed with a Nikon confocal live cell imaging system for 48 hours, and quantitative results were analyzed using GraphPad Prism 8 software.

### Xenograft Mouse Model

NOD.CB17/Prkdcscid/JKrb/ IL2 receptor γ^-/-^(NIG) mice 6 weeks old, which are highly immunodeficient mice, were obtained from GHBIO Inc. (Genes to Health Biotechnology, Daejeon, Korea). Mouse xenograft models were generated by subcutaneous injection of 1 × 10^7^ K562 tumor cells with Matrigel (Corning) in the left flank of female NIG mice once a week for a total of 3 doses. For the evaluation of anti-tumor efficacy, NK-92 cells, NK-92 cells with recombinant human IL-2 (rhIL-2) or MBP NK cells were intravenously injected into the K562 xenograft mice model into the tail vein at a concentration of 5 × 10^6^ cells a head on day 7 after inoculation with the K562 tumor cell line. For NK-92 with the rhIL-2 group, 1000 U of rhIL-2 was administered every other day. Tumor sizes were calculated every 2 or 3 days with a caliper for a period of four weeks by the following equation: tumor volume = W^2^ × L / 2 (W is tumor width and L is tumor length). For all animals, the body weight was measured immediately before the start of every next NK-cell infusion by an electronic scale. To measure human interferon-γ (hIFN-γ) in mouse plasma, blood from all animals was collected in an EDTA (anti-coagulant)-treated storage container at the time of autopsy and plasma was separated by centrifugation.

### Ethical Approval

*In vivo* tumor growth inhibition tests in mouse models were performed following protocols approved by the Institutional Animal Care and Use Committee (IACUC) of GH Bio, Korea (Approval number, AE2021-33).

### Statistical Analysis

All results presented in graphs are shown as the mean value ± S.D. from over three independent experiments and statistically analyzed with an unpaired two-tailed t-test. Asterisks represent statistically significant differences (*ρ < 0.05, **ρ < 0.01, ***ρ < 0.001).

## Supplementary Material

Supplementary figures, table, method.

Supplementary video 1.

Supplementary video 2.

Supplementary video 3.

Supplementary video 4.

Supplementary video 5.

Supplementary video 6.

Supplementary video 7.

Supplementary video 8.
