# Supplementary material for: Cytokine engineered NK-92 therapy to improve persistence and anti-tumor activity
Source: Theranostics. 2023 Mar 5;13(5):1506–19. doi: 10.7150/thno.79942 (PMC10086201; doi:10.7150/thno.79942)
Supplement: Supplementary file 1 — Supplementary figures, table, method. [file thnov13p1506s1.pdf]

Supporting Information

Cytokine engineered NK-92 therapy to improve persistence and anti-tumor activity

Hyun Young Shin[1], Seil Jang[1], Hyeong Jung Woo[2], Jae-Hee Chung[1], Woon-Hae Kim[1], Dongoh Kim[1], Minju Kang[1], Yujin Lim[1], Omer Habib[1], Jungmin Lee[1], Sohae Yang[2], Dae Hee Lee[1], and Minseok S. Kim[1,2,3,4]*

1. CTCELLS Inc., 216, Gaepo-ro, Gangnam-gu, Seoul, 06307, Republic of Korea
2. Department of New Biology, DGIST, Daegu, 42988, Republic of Korea
3. Translational Responsive Medicine Center (TRMC), DGIST, Daegu 42988, Republic of Korea
4. New Biology Research Center (NBRC), DGIST, Daegu 42988, Republic of Korea

* Corresponding authors: Minseok S. Kim, Department of New Biology, DGIST, Daegu 42988, Republic of Korea. E-mail: kms@dgist.ac.kr

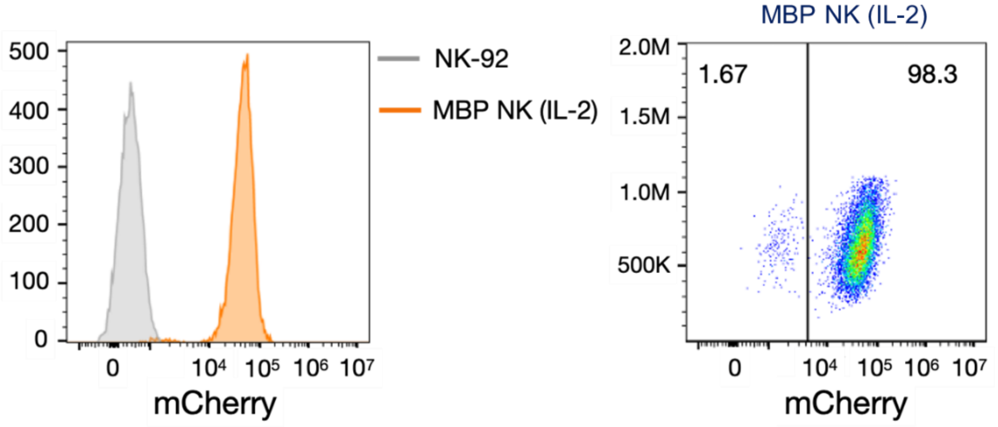

Figure S1. Membrane-bound IL-2 expression in the MBP NK cells

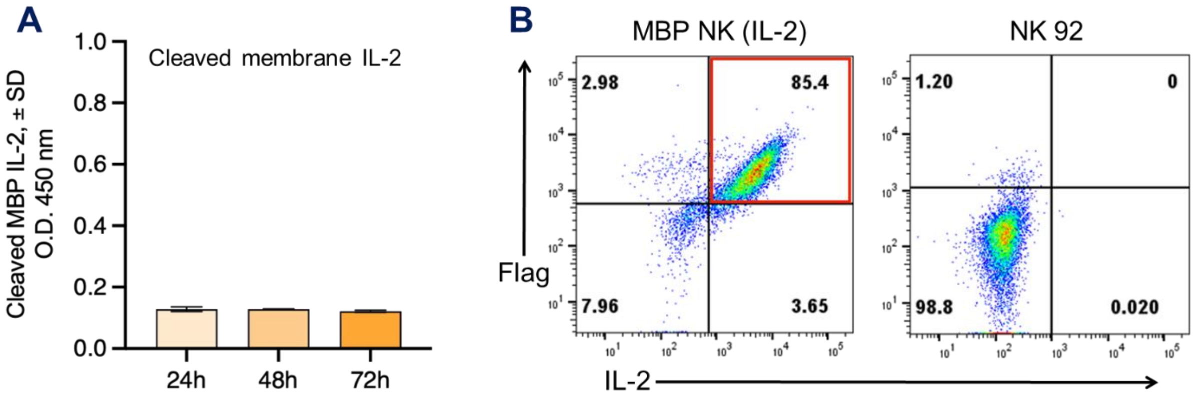

Figure S2. Stability of membrane bound IL-2 in MBP NK. **(A)** ELISA was introduced to investigate the cleaved form of membrane bound IL-2 in the MBP NK culture medium at specific time points (24, 48, and 72 hrs). Triplicate determinations are shown as Means ± SDs. **(B)** The flow cytometry analysis shows that MBP IL-2 can be detected on the surface of the cells via the anti-flag as well as anti-IL-2 antibodies.

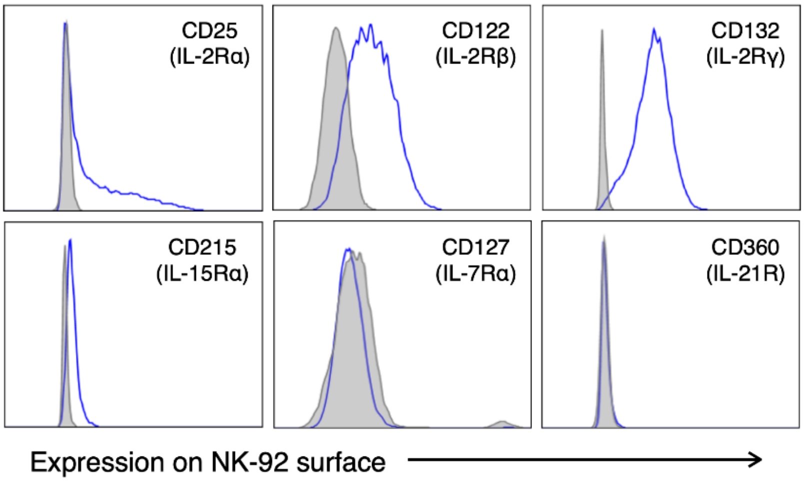

Figure S3. The cytokine receptors of NK-92 cells. The presence of cytokine receptors on the surface of NK-92 cells was analyzed by the flow cytometry system with following antibodies: APC-conjugated anti-CD25 antibody, PE-cyanine7-conjugated anti-CD122 antibody, PE-conjugated anti-CD132 antibody, PE-conjugated anti-CD215 antibody, PE-cyanine7-conjugated anti-CD127 antibody and APC-conjugated anti-CD360 antibody. IL-2 receptors were more abundant compared to IL-15, IL-7 or IL-21 receptor.

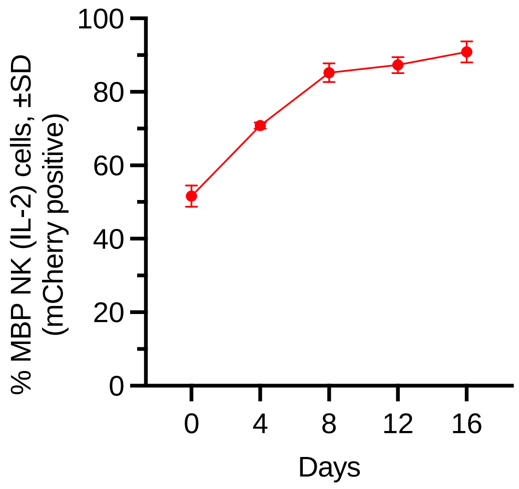

Figure S4. The change in the population of MBP NK (IL-2) cells during co-culture with NK-92 cells in the absence of exogeneous IL-2. The ratio of NK-92 cells to MBP NK (IL-2) cells was 1 : 1 and co-cultured for 8 days without IL-2. The percentage of MBP NK (IL-2) cells was analyzed using flow cytometry by mCherry detection.

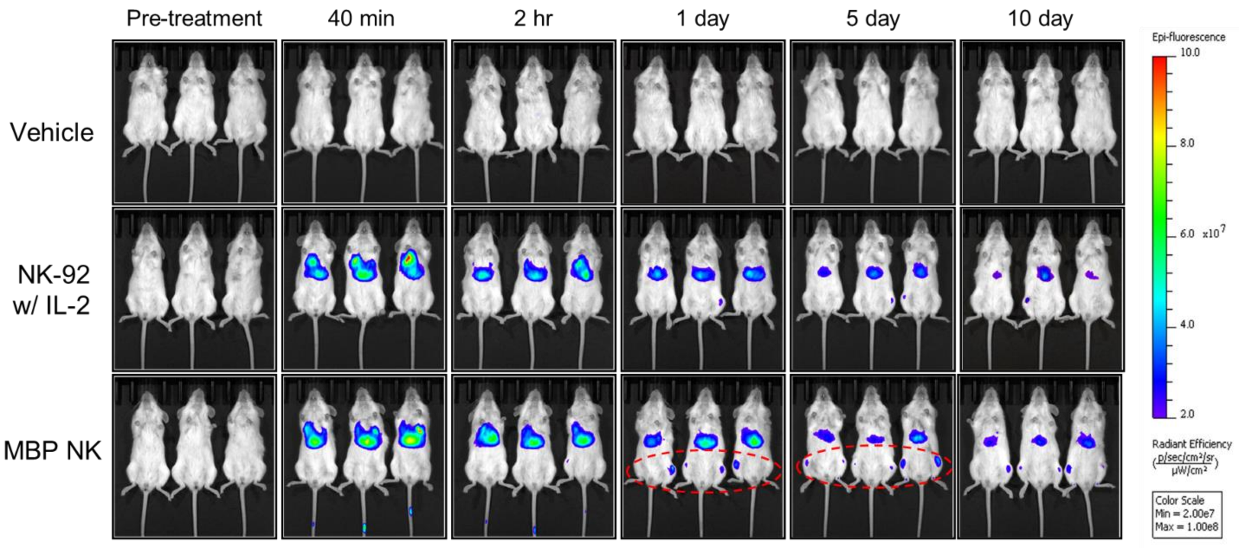

Figure S5. Biodistribution of MBP NK *in vivo*. The IVIS Spectrum was used for detection of DIR 750 labeled MBP NK or NK-92 following infusion via the tail vain of mice. The measurement of radiance of DIR 750 labeled NK-92 or MBP NK from each animal was detected at specific time points (0, 40 min, 2 h, D1, D5, and D10) using IVIS Spectrum.

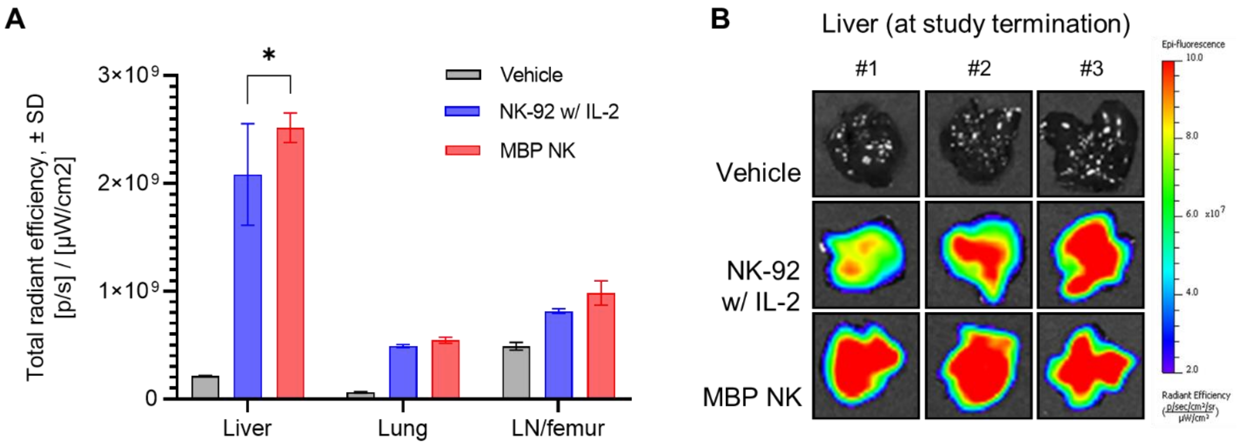

Figure S6. Long-term persistence of the MBP NK *in vivo*. **(A)** Liver, lungs, and LN/femur were isolated from mice and analyzed for measuring the intensity of organ-resident DIR 750 labeled MBP NK and NK-92 at the end of experiment. Data were reported as mean ± SD and statistical significance was determined by the one-way ANOVA with Dunnett's multiple comparison test. *, $p = 0.0105$. **(B)** Images of the fluorescence intensity of the liver were taken.

Table S1. Antibody list used for surface marker analysis

No	Markers	Isotype	Fluorescence	Clone	Company
1	CD56	Mouse IgG1	Alexa Fluor488	5.1H11	Biolegend
2	NKp30	Mouse IgG1	Alexa Fluor647	P30-15	Biolegend
3	H7-B6	Mouse IgG1	PE	875001	R&D system
4	CD25 (IL-2Rα)	Mouse IgG1	APC	3G10	MACS
5	CD122 (IL-2Rβ)	Mouse IgG1	PE-cyanine7	TU27	Biolegend
6	CD132 (IL-2Rγ)	Rat IgG2b	PE	TUGh4	Biolegend
7	CD215 (IL-15Rα)	Rat IgG2b	PE	JM7A4	Biolegend
8	CD127 (IL-7Rα)	Mouse IgG1	PE-cyanine7	A019D5	Biolegend
9	CD360 (IL-21Rα)	Rat IgG2a	APC	W18100A	Biolegend
10	Isotype control	Mouse IgG1	Alexa Fluor488	MOPC-21	Biolegend
11		Mouse IgG1	PE	MOPC-21	Biolegend
12		Mouse IgG1	Alexa Fluor647	MOPC-21	Biolegend
13		Mouse IgG1	APC	MOPC-21	Biolegend
14		Mouse IgG1	PE-cyanine7	MOPC-21	Biolegend
15		Rat IgG2a	APC	RTK2758	Biolegend
16		Rat IgG2b	PE	RTK4530	Biolegend

Supplementary Method

Analysis of Degranulation (CD107a)

The NK cells were co-cultured with K562 cells at a ratio 1:1 for 4 hours, added PE-conjugated anti-CD107a antibody (R&D systems) at the beginning of the co-cultures. After 1 hour of the cocultures, GolgiStop^{TM} (BD Biosciences) was added. The percentage of CD107a+ NK cells was determined by flow cytometry, and the result was analyzed using GraphPad Prism 8 software.
